# Specific IgE to tropomyosin increases the diagnostic accuracy of shrimp allergy

**DOI:** 10.3389/falgy.2026.1737009

**Published:** 2026-01-23

**Authors:** Prapasri Kulalert, Piyawut Kreetapirom, Surapon Piboonpocanun, Orathai Jirapongsananuruk, Nattakan Authong, Nantika Khodtecha, Orapan Poachanukoon, Sira Nanthapisal

**Affiliations:** 1 Department of Clinical Epidemiology, Faculty of Medicine, Thammasat University, Pathum Thani, Thailand; 2 Center of Excellence for Allergy, Asthma and Pulmonary Disease, Thammasat University, Pathum Thani, Thailand; 3 Division of Pediatrics, Chaophya Hospital, Bangkok, Thailand; 4 Institute of Molecular Biosciences, Mahidol University, Nakhon Pathom, Thailand; 5 Division of Allergy and Clinical Immunology, Department of Pediatrics, Faculty of Medicine Siriraj Hospital, Mahidol University, Bangkok, Thailand; 6 Department of Pediatrics, Faculty of Medicine, Thammasat University, Pathum Thani, Thailand

**Keywords:** component-resolved diagnosis, diagnosis accuracy, oral food challenge, shrimp allergy, tropomyosin (TPM)

## Abstract

**Purpose:**

Skin prick test (SPT) and the level of serum-specific IgE (sIgE) antibodies to shrimp have low specificity in the diagnosis of shrimp allergy. Measurement of sIgE to tropomyosin is available as a test, but the accuracy remains controversial. This study aims to evaluate the diagnostic accuracy of sIgE measurement to tropomyosin in the diagnosis of shrimp allergy and compare the diagnostic performance to SPT and sIgE to shrimp.

**Methods:**

Patients with a history of immediate reaction to shrimp allergy were recruited. All participants underwent SPT with commercial shrimp extract. Measurements of sIgE to shrimp and tropomyosin were carried out. An oral food challenge (OFC) with shrimp was performed to confirm the diagnosis.

**Results:**

Fifty symptomatic patients (mean age 27.3 years) with suspected shrimp allergy were evaluated. OFC confirmed allergy in 13 (26%) patients. Diagnostic modalities offered distinct advantages and limitations. Tropomyosin sIgE (rPen a 1) yielded superior specificity (91.9%) at the cost of sensitivity (23.1%), while extract-based tests (SPT and shrimp sIgE) provided better sensitivity (46.2%–61.5%) but lacked specificity (43.2%–51.4%). Implementing a two-step algorithm—combining SPT with tropomyosin sIgE—successfully optimized specificity to 94.6%. Nevertheless, given the suboptimal predictive values across all methods (PPV 25%–50%; NPV 73%–77%), these tools alone cannot safely guide management, and OFC remains essential.

**Conclusion:**

Measurements of sIgE to tropomyosin provided higher specificity and increased diagnostic efficiency than SPT and measurement of sIgE to shrimp for the diagnosis of shrimp allergy.

## Introduction

Shrimp is a common cause of food allergy (1, 2). Symptoms can occur in a single organ such as the skin (e.g., urticaria and lip/tongue swelling), respiratory system (e.g., chest tightening and wheezing), gastrointestinal system (e.g., oral allergy syndrome and vomiting), or cardiovascular system (e.g., low blood pressure). In some cases, multiple organ system involvement occurs such as anaphylaxis in which urticaria and angioedema are the most common accompanying symptoms (3, 4). These reactions usually occur within 2 h after ingestion and involve an IgE-mediated mechanism.

Similar to other food allergies, the conventional investigation tools for immediate-type hypersensitivity reactions to shrimp are skin prick test (SPT), serum-specific IgE (sIgE) for food measurement, or both. Oral food challenge (OFC) is the gold standard tool to exclude or confirm a diagnosis but should be performed under specialist supervision because this procedure might induce a severe allergic reaction (5). However, SPT with shrimp commercial extracts and measurements of sIgE to shrimp have shown low specificities of <50% for a shrimp allergy diagnosis (6, 7). Hence, the definite diagnosis for shrimp allergy remains challenging.

An understanding of allergens, including their specific IgE-binding epitopes, and improvements in technology to produce recombinant allergens allow the development of component-resolved diagnosis (CRD). CRD detects IgE antibodies specific to entire allergenic proteins (components), providing a more precise diagnosis than whole extracts (8). CRD testing has demonstrated high specificity for food allergy diagnosis (e.g., cow's milk and egg) (9). However, reports on using CRD in the diagnosis of shrimp allergy are lacking.

Tropomyosin has been identified as the major shrimp allergen in several shrimp species including *Penaeus aztecus* (Pen a 1), *Penaeus indicus* (Pen i 1), *Penaeus monodon* (Pen m 1), *Metapenaeus ensis* (Met e 1), and *Litopenaeus vanamei* (Lit v1) (10, 11). Tropomyosin was shown to share immunodominant epitopes among all species of crustaceans (12). *In silico* analyses of Lit v 1 show numerous high-identity matches with tropomyosins from multiple shrimp and prawn species (13). A commercial test for measurements of serum IgE to recombinant tropomyosin (rPen a 1) is currently available. However, the accuracy remains controversial. Gámez et al. (14) reported a specificity of 77% for sIgE levels to rPen a 1. In contrast, Ayuso et al. (15) reported a specificity of only 45.5%. One significant factor contributing to the variation in shrimp sensitization is house dust mite (HDM) allergy. This is due to the cross-reactivity between tropomyosin in shrimp and house dust mite (16). It is important to note that Thailand is an HDM-endemic area (17). A small study in this population reported that 30% of patients with shrimp allergy had positive sensitization to HDM (18).

We aimed to evaluate the diagnostic accuracy of measuring sIgE antibodies to tropomyosin in the diagnosis of *Litopenaeus vanamei* shrimp allergy. We also aimed to compare its diagnostic performance with the results of SPT and the levels of serum-specific IgE antibody to shrimp using commercial testing and recombinant *Litopenaeus vanamei* IgE testing.

## Materials and methods

### Study participants

Patients with a history of immediate reaction to shrimp were recruited into a cross-sectional study; all participants were followed up at the Thammasat University Center of Excellence for Allergy, Asthma, and Pulmonary Disease. The immediate reaction was defined as ≥1 reported symptom of skin (e.g., urticaria and lip/tongue swelling), respiratory system (e.g., chest tightening and wheezing), gastrointestinal system (e.g., vomiting), or cardiovascular system (e.g., low blood pressure), which occurred within 2 h after consumption (19).

Patients with a history of delayed reactions or only subjective symptoms (e.g., pruritus) were excluded. We also excluded patients with an underlying disease, such as cardiovascular disease, or patients who did not complete the allergy testing.

The study was approved by the Institutional Review Board and the Ethics Committee of the Faculty of Medicine, Thammasat University. Written informed consent was obtained from the patients or parents if the patients were ≤18 years of age.

### Skin prick test

SPT was performed with commercial shrimp extracts (ALK-Abello, New York, USA). Histamine 1 mg/mL and 50% glycerin were used as positive and negative controls, respectively. One drop of each reagent was applied to the volar surface of the forearm using a 1 mm single peak lancet by experienced nurses and read after 15‒20 min. The SPT result was defined as positive if the mean wheal diameter was ≥3 mm.

### Measurement of IgE antibodies to shrimp and tropomyosin

Serum IgE to shrimp was measured using the f24 ImmunoCAP (Thermo Fisher Scientific, Uppsala, Sweden). The f24 ImmunoCAP provides a quantitative measure of specific IgE using an extract prepared from the following species: *Penaeus monodon*, *Metapenaeopsis barbata*, *Pandalus borealis*, and *Metapenaeus joyneri*. Specific IgE to tropomyosin was measured using a f351 recombinant Pen a 1 (rPen a 1; Thermo Fisher Scientific, Uppsala, Sweden). Serum IgE to shrimp and tropomyosin levels were considered positive if the level was ≥0.35 kUA/L.

### Oral food challenge

The OFC was performed by administering increasing doses of 1, 2, 4, 8, 16, and 32 g of boiled shrimp (*Litopenaeus vanamei)* at 15–20 min intervals with a total amount of 63 g equal to approximately 15 g of shrimp protein (6). The OFC was performed and assessed by a team of experienced nurses and allergists. Emergency medications, such as epinephrine, antihistamine, steroids, and equipment, were prepared. During the OFC, the patients were monitored for vital signs, abnormal signs, and symptoms (e.g., urticaria and angioedema) before escalating each dose.

The OFC was discontinued if participants developed objective signs of IgE-mediated allergic reactions, in accordance with the well-established guidelines (20). A positive challenge required objective involvement of the skin, respiratory, gastrointestinal, or cardiovascular systems. Stopping criteria included the following: (1) generalized urticaria or progressive angioedema, (2) respiratory compromise (persistent cough, wheeze, stridor, voice change, dyspnea, or ≥20% reduction in peak expiratory flow), (3) repetitive vomiting or severe abdominal pain, or (4) cardiovascular compromise (hypotension or syncope). Anaphylaxis or persistent/recurrent objective symptoms affecting ≥2 organ systems were also considered positive. Isolated, mild, and transient symptoms (e.g., limited erythema or oral pruritus) were recorded but not considered sufficient to halt the test. Isolated throat tightness without objective signs led to reassessment and temporary delay only; the challenge was stopped and considered positive if symptoms persisted or progressed. Participants were observed for at least 2 h after the final dose.

### Statistical analysis

Data were analyzed using STATA version 14.0. Demographic characteristics were described as numbers and percentages for categorical data, mean ± standard deviation (SD), or median interquartile range for continuous data, as appropriate. The diagnostic accuracy was calculated in terms of sensitivity, specificity, positive predictive value (PPV), negative predictive value (NPV), and efficiency. Efficiency was defined as the proportion of true positive and true negative results detected among the total number of participants who performed the test.

## Results

### Patient characteristics

Fifty patients participated in this study. The mean ± SD age was 27.3 ± 10.8 years, and the number of females was 36 (72.0%). The mucocutaneous symptoms including angioedema (60%) and urticaria (58%) were the most common clinical presentation, followed by respiratory symptoms (e.g., chest tightness; 40%) and gastrointestinal symptoms (e.g., vomiting; 18%), respectively (Table 1). Half of the patients reported mucocutaneous symptoms only while the other half reported multiple organ system symptoms. The presenting symptoms and demographics are presented in Table 1.

**Table 1:** Characteristics of the study population.

Characteristics	
Mean age (years), mean ± SD	27.3 ± 10.8
Gender	
Female	36 (72.0)
Male	14 (28.0)
Atopic history	
Asthma	3 (6.0)
Allergic rhinitis	23 (46.0)
Atopic dermatitis	8 (16.0)
Other food allergy	20 (40.0)
Crab	16
Squid	2
Clams	2
Wheat	2
Clinical manifestations^a^	
Skin/oral/mucosal tissue symptoms	
Urticaria	29 (58.0)
Angioedema	30 (60.0)
Itching in the oral cavity	27 (54.0)
Respiratory symptoms	
Nasal symptoms	8 (16.0)
Chest tightness	20 (40.0)
Gastrointestinal symptoms	
Vomiting	9 (18.0)
Diarrhea	5 (10.0)
Single organ	
Cutaneous system	25 (50.0)
Multiple organs	
Cutaneous, respiratory system	14 (28.0)
Cutaneous, gastrointestinal system	4 (8.0)
Respiratory, gastrointestinal system	1 (2.0)
Cutaneous, respiratory, gastrointestinal system	6 (12.00)

Data are presented as *n* (%) unless otherwise indicated.

SD, standard deviation.

a Some patients reported >1 symptoms.

### Diagnostic performance of different allergy tests

Oral challenges confirmed the diagnosis of shrimp allergy in 13 patients (26.0%). The results of SPT, sIgE to shrimp, and sIgE to tropomyosin are summarized in Table 2, and the diagnostic performance of each allergy test is reported in Table 3. From the total of 50 patients, 6 of the 13 patients (46.2%) who had a confirmed shrimp allergy diagnosis had positive SPT results. In the tolerant group, 18 of the 37 patients (48.7%) had positive SPT results. While these results indicate immunological sensitization, they were not associated with clinical reactivity following the oral food challenge using the specific shrimp material and dose protocol utilized in this study. The results of the SPT revealed that the sensitivity, specifically, PPV, and NPV were 46.2%, 51.4%, 25.0%, and 73.1%, respectively.

**Table 2:** Results of skin prick test, sIgE to shrimp, and sIgE to tropomyosin (*N* = 50).

Allergy testing and results	Allergic group (*n* = 13)	Tolerant group (*n* = 37)	*P*-value
Skin prick test			
Positive	6 (46.2%)	18 (48.6%)	
Negative	7 (53.8%)	19 (51.4%)	
Wheal size (mm)			
Median [IQR]	0 [0, 4.99]	2.65 [0, 4.72]	0.870
Min–Max	0–12.46	0–10.20	
sIgE to shrimp (f24)			
Positive	8 (61.5%)	21 (56.8%)	
Negative	5 (38.5%)	16 (43.2%)	
Level (kUA/L)			
Median [IQR]	0.77 [0.16, 2.12]	0.58 [0.1, 2.35]	0.550
Min–Max	0.04–33.5	0–36	
sIgE to tropomyosin (f351)			
Positive	3 (23.1%)	3 (8.1%)	
Negative	10 (76.9%)	34 (91.9%)	
Level (kUA/L)			
Median [IQR]	0.01 [0,0.08]	0.02 [0,0.04]	0.767
Min–Max	0–5.05	0–2.34	

IQR, interquartile range; SPT, skin prick test.

**Table 3:** Diagnostic accuracy of skin prick test, sIgE to shrimp, or sIgE to tropomyosin alone and the combination of tests.

Allergy testing	Sensitivity (%)	Specificity (%)	PPV (%)	NPV (%)	Efficiency (%)	AUC
Single test						
Skin prick test (SPT)	46.2%	51.4%	25.0%	73.1%	50.0%	0.49 (0.32–0.65)
sIgE to shrimp	61.5%	43.2%	27.6%	76.2%	48.0%	0.52 (0.36–0.68)
sIgE to tropomyosin	23.1%	91.9%	50.0%	77.3%	74.0%	0.57 (0.45–0.70)
Combination test^a^						
SPT, sIgE to shrimp	46.2%	70.3%	35.3%	78.8%	64.0%	0.58 (0.42–0.74)
SPT, sIgE to tropomyosin	23.1%	94.6%	60.0%	77.8%	76.0%	0.59 (0.46–0.71)
sIgE to shrimp, tropomyosin	23.1%	91.9%	50.0%	77.3%	74.0%	0.57 (0.45–0.70)

NPV, negative predictive value; PPV, positive predictive value; SPT, skin prick test.

a Test positive was defined as positive to both tests.

Twenty-nine patients had levels of sIgE to shrimp ≥0.35 kUA/L; however, only eight subjects (27.6%) were confirmed to be allergic to shrimp by OFC. More than 50% of patients passed the OFC despite sIgE to shrimp ≥0.35 kUA/L. Wheal size from skin testing, sIgE to shrimp, and tropomyosin were not statistically different between the allergic and tolerant group (Supplementary Table S1). The sensitivity, specificity, PPV, and NPV of sIgE antibodies to shrimp were 61.5%, 43.2%, 27.6%, and 76.2%, respectively.

Three patients (23.1%) of the 13 positive OFC patients had positive levels of sIgE to rPen a 1 (≥0.35 kUA/L). Interestingly, among the 37 patients who could tolerate shrimp, 34 (91.9%) had negative sIgE to tropomyosin results. Only 3 (8.1%) patients appeared to have had false-negative test results as they developed reactions during the OFC. Hence, the specificity of sIgE to rPen a 1 from our findings was 91.9%, sensitivity of 23.1%, PPV of 50.0% for PPV, and NPV of 77.3%. We also evaluated alternative cutoff values for shrimp and tropomyosin-specific IgE, but none improved diagnostic performance. ROC analysis failed to identify a clinically meaningful threshold, as AUC values remained low and unchanged across different cutoffs, reflecting inherent limitations of these biomarkers rather than cutoff selection. Therefore, the conventional cutoff of ≥0.35 kUA/L was retained for consistency and comparability (Supplementary Tables S1, S2).

Finally, measurement of sIgE to tropomyosin gives the highest diagnostic efficiency (74.0%) followed by SPT (50.0%) and measurement of sIgE to shrimp (48.0%).

### Diagnostic performance of SPT combined with sIgE to tropomyosin

**Figure 1:**
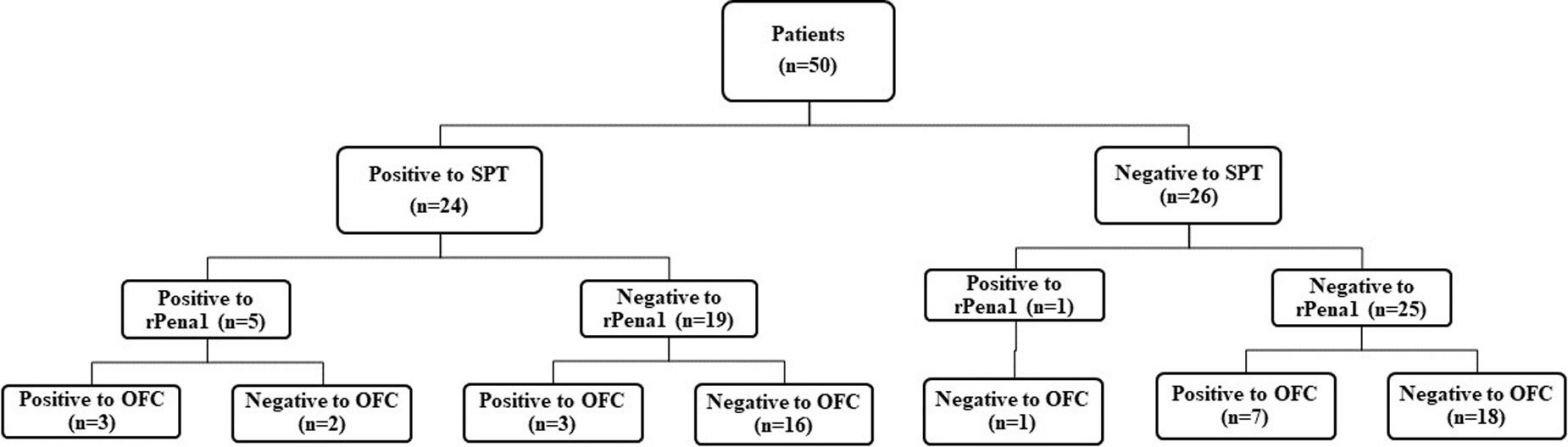
Diagnostic performance of skin prick testing to shrimp commercial extract in the combination with specific IgE to tropomyosin (rPen a1) OFC, oral food challenge; rPen a1, recombinant *Penaeus aztecus* tropomyosin; SPT, skin prick testing.

Figure 1 shows the two-step diagnostic approach for shrimp allergy using SPT and sIgE level to tropomyosin. A total of 24 patients had positive results of SPT. When sIgE to tropomyosin was undertaken as the second test, 16 of 24 patients (66.7%) had positive results of SPT but had negative results of sIgE to tropomyosin in which all of 16 patients were tolerant to shrimp (negative OFC). Therefore, sIgE to tropomyosin is useful to decrease false-positive results for SPT in >50% of cases.

The diagnostic performance of the combination of SPT and sIgE to tropomyosin is shown in Table 3. Test positive was defined as positive to both SPT and sIgE to tropomyosin. The combination of the test results improved the diagnostic performance of the specificity (94.6%) compared with either the SPT (51.4%) or sIgE to shrimp tropomyosin (91.9%) alone.

### Diagnostic performance of sIgE to shrimp and sIgE to tropomyosin

**Figure 2:**
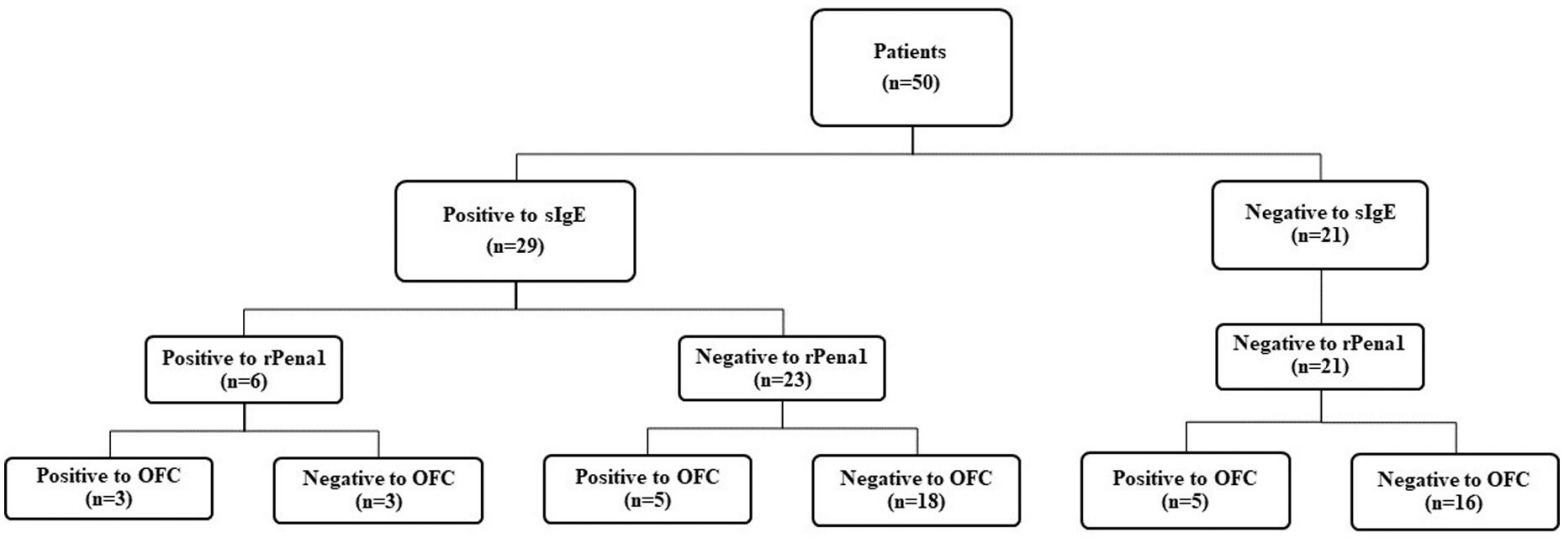
Diagnostic performance of specific IgE to shrimp in the combination with specific IgE to tropomyosin (rPen a1) OFC, oral food challenge; rPen a1, recombinant *Penaeus aztecus* tropomyosin; SPT, skin prick testing.

**Figure 3:**
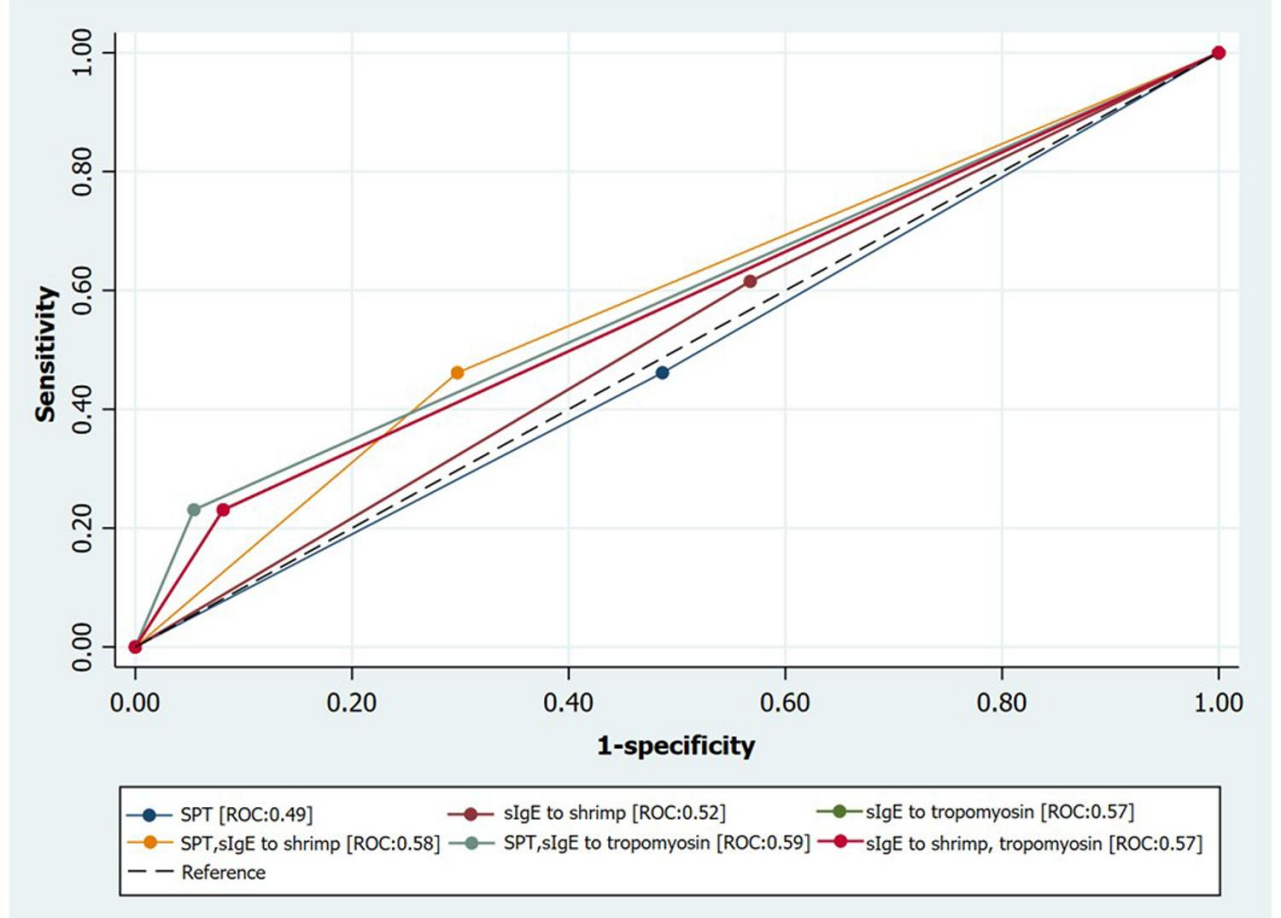
Receiver operating characteristic (ROC) curves illustrating the distribution area under curve values for each diagnostic test: sIgE, specific immunoglobulin E; SPT, skin prick testing.

The stepwise diagnostic approach algorithm for sIgE to shrimp and sIgE to tropomyosin is shown in Figure 2. From a total of 50 patients, 29 patients showed positive sIgE to shrimp. When sIgE to tropomyosin was undertaken as the second test, 18 of the 29 patients (62.1%) had negative results of sIgE to tropomyosin, and they were also tolerant to shrimp. Therefore, adding sIgE to tropomyosin as the second investigation decreases false-positive results for sIgE to shrimp. The specificity, sensitivity, PPV, and NPV after combining sIgE to shrimp and tropomyosin were 91.9%, 23.1%, 50.0%, and 77.3%, respectively (Table 3). Figure 3 also illustrates the receiver operating characteristic curve representing the distribution of area under the curve values for each single and combined diagnostic test.

## Discussion

The diagnosis of shrimp allergy remains challenging despite its high prevalence. A key difficulty lies in the complex and species-specific variability of the major allergen, tropomyosin, which contributes to heterogeneity in IgE immune responses. Currently available diagnostic tools, including commercial skin prick testing (SPT) and serum shrimp-specific IgE (sIgE) assays (f24; ImmunoCAP), rely on crude shrimp extracts. The commercial shrimp extract used for SPT (ALK-Abello) is prepared from *Litopenaeus setiferus*, a prawn commonly found along the Atlantic coast. Meanwhile, the shrimp-specific IgE assay (f24 ImmunoCAP) contains a mixture of shrimp species, typically including *Pandalus borealis* (cold-water species) as well as *Penaeus* and *Metapenaeus* species (warm-water Indo-Pacific shrimp widely consumed worldwide). These extract-based tests primarily reflect sensitization rather than true clinical allergy and have limited ability to predict reactivity or reaction severity to specific shrimp species. Consequently, OFC with a defined shrimp species remains necessary for definitive diagnosis. To improve diagnostic accuracy, a multitiered approach is increasingly advocated. While sensitive crude extracts serve as effective screens, adding tropomyosin assessment (CRD) significantly enhances specificity and predicts clinical reactivity (14, 21, 22).

In the present study, sIgE to tropomyosin demonstrated the highest specificity, followed by SPT to commercial shrimp extract and whole shrimp sIgE. Diagnostic efficiency was also superior for sIgE to tropomyosin compared with the other modalities. In Thailand, tropomyosin has been identified as a major allergen among shrimp-allergic patients to both *Penaeus monodon* and *Macrobrachium rosenbergii* (23, 24). However, component sensitization patterns show geographic variability (21, 25–29). In European and some non-Asian cohorts, tropomyosin is not always the dominant component; instead, other components—such as sarcoplasmic calcium-binding protein (SCP), myosin light chain (Lit v 3), arginine kinase (AK), and other high-molecular-weight proteins—appear to play a more prominent role. In such settings, tropomyosin-specific IgE alone demonstrates lower diagnostic accuracy and is no longer considered a sufficient standalone biomarker (28–30). Therefore, the high specificity of sIgE to tropomyosin and its combination with SPT observed in our Thai cohort should be interpreted as context-specific and may not be directly generalizable to regions with different sensitization profiles.

Our results are consistent with previous studies. Yang et al. (22) reported that the specificity of sIgE to tropomyosin (92.8%) was greater than sIgE to shrimp (75%) and SPT to shrimp extract (64.2%). Serum IgE to tropomyosin had greater diagnostic efficiency (88.5%) compared with whole shrimp-specific IgE (74.2%) or SPT (65.7%). Wai et al. (31) reported low specificities of 28% and 35% for SPT and sIgE to shrimp, respectively, but the specificity of sIgE to rPen a 1 was 90%. Similarly, the results of Thalayasingam et al. (7) demonstrated that SPT using shrimp commercial extract and sIgE to shrimp had specificities of only 37% and 50%, respectively, but the specificity of sIgE to tropomyosin was 85.2%.

For this reason, component-resolved diagnostics (CRD) are recommended as a second-line test. Patients with positive SPT but negative component testing should undergo OFC to confirm or exclude true clinical allergy.

In conventional clinical practice, SPT is usually the first-line investigation. Patients with positive results are frequently advised to avoid shrimp without confirmatory testing. Although prick-to-prick testing using cooked shrimp has been shown to yield high specificity—up to 100% when performed with *Penaeus monodon* or *Macrobrachium rosenbergii*—there is a small but definite risk of systemic reactions (∼0.02%), particularly in high-risk patients (6). For this reason, component-resolved diagnostics (CRD) are recommended as a second-line test (32). Patients with positive SPT but negative component testing should undergo OFC to confirm or exclude true clinical allergy.

In our cohort, tropomyosin-specific IgE demonstrated superior specificity (91.9%) compared with extract-based tests; PPVs remained suboptimal (<50%), likely due to low disease prevalence (26%) and potential HDM cross-reactivity (Der p 10 was not assessed). However, combining SPT with sIgE to tropomyosin (rPen a 1) increased diagnostic specificity to 95%. In contrast, combining SPT with shrimp sIgE only modestly improved specificity (70.3%) and remained limited by low sensitivity (46.2%) and moderate NPV (78.8%). Clinically, this implies that nearly one-quarter of patients with negative results on both SPT and shrimp sIgE were still allergic by OFC. Therefore, this strategy cannot safely exclude shrimp allergy in patients with a convincing history. Our results support prior evidence that extract-based SPT and shrimp sIgE have limited predictive value and cannot replace OFC, particularly in high-suspicion cases (5, 7, 10, 16, 27).

The high specificity we observed for combined SPT and sIgE to tropomyosin suggests that this approach may be useful for reducing false-positive diagnoses and unnecessary dietary avoidance. However, it should be noted that our OFC protocol used a cumulative dose of 63 g of boiled shrimp (equivalent to approximately 15 g of shrimp protein), corresponding to a typical serving in Thailand. This dose lies at the lower end of the recommended 15–20 g range (20). Testing with lower challenge doses may increase the risk of false-negative results in individuals with higher reaction thresholds or those requiring specific co-factors (e.g., exercise and NSAIDs) to elicit symptoms. Thus, residual misclassification cannot be completely excluded.

A critical limitation observed across all diagnostic modalities in our study—including SPT, extract-sIgE, and tropomyosin sIgE—was the uniformly poor sensitivity and negative predictive values (NPV). Specifically, tropomyosin sIgE showed a sensitivity of only 23%, meaning it failed to identify nearly 80% of true allergy cases. This finding is in line with an Italian multicenter study, which reported Pen a 1 sensitization in 41% of shrimp-allergic adults (25). The low sensitivity of tropomyosin highlights the relevance of ‘context-specific’ allergen profiles. Sensitization patterns vary significantly by geography and consumption habits. While tropomyosin is often the major allergen in mite-sensitized populations, our data suggest that our cohort may be predominantly sensitized to other heat-stable or labile components not captured by this single test. Minor allergens such as AK, SCP, and myosin light chain are increasingly recognized as clinically relevant triggers, particularly in Asian populations (21, 24, 26–28). Recent studies further indicate that tropomyosin alone is insufficient as a diagnostic marker; recent studies indicate that combining multiple recombinant components (e.g., AK and SCP alongside tropomyosin) can significantly improve diagnostic sensitivity (29). Future research should therefore focus on evaluating multicomponent panels for more accurate diagnosis and tailored to local sensitization profiles to reduce the reliance on OFC.

Several limitations must be acknowledged. First, we employed an open OFC design rather than a double-blind, placebo-controlled OFC, which is considered the gold standard. However, the open method is more feasible in routine practice, and we minimized bias by defining positivity based on objective clinical signs. Moreover, nurses and allergists conducting the OFC were blinded to all index test results. Second, there was a species mismatch between diagnostic tests and the challenge material. The commercial SPT and f24 ImmunoCAP extracts contain mixed shrimp species (e.g., *P. monodon*, *M. barbata*, *P. borealis*, *M. joyneri*), whereas the OFC used *Litopenaeus vannamei*. Additionally, the tropomyosin assay employed recombinant Pen a 1 from *P. aztecus*. *In silico* analysis by Soto-Muñoz et al. (33) showed very high amino acid sequence identity (95%–100%) between tropomyosin from *L. vannamei* and *P. aztecus*, supporting the potential for IgE cross-reactivity. Nevertheless, sequence homology does not guarantee identical allergen expression or clinical relevance, and this remains a methodological limitation. Third, due to the retrospective nature of clinical history taking, the specific shrimp species responsible for the index reaction could not be reliably determined. Given the recognized phenomenon of species-specific prawn allergy, this limitation may partly explain discordance between clinical history and OFC results. Finally, the relatively small number of OFC-positive patients (*n* = 13) reduces statistical precision and results in wider confidence intervals for sensitivity, PPV, and NPV estimates. These findings therefore require confirmation in larger prospective studies.

In conclusion, conventional diagnostic tools for shrimp allergy—SPT and shrimp sIgE—demonstrate low specificity and limited clinical utility. Measurement of tropomyosin-specific IgE offers significantly higher specificity and improved diagnostic efficiency, either alone or when combined with SPT. This approach may reduce false-positive diagnoses and unnecessary dietary restriction, although OFC remains essential for definitive diagnosis, particularly in patients with compatible clinical histories.

## Data Availability

The raw data supporting the conclusions of this article will be made available by the authors, without undue reservation.
