# Supplementary material for: Specific IgE to tropomyosin increases the diagnostic accuracy of shrimp allergy
Source: Front Allergy. 2026 Jan 23;7:1737009. doi: 10.3389/falgy.2026.1737009 (PMC12876172; doi:10.3389/falgy.2026.1737009)
Supplement: Supplementary file 1 [file Table1.docx]

**Supplementary Table 1** Diagnostic accuracy of sIgE to shrimp at each cut-off point

|  | **Sensitivity (%)** | **Specificity (%)** | **PPV (%)** | **NPV (%)** | **Efficiency (%)** | **AUC** |
| --- | --- | --- | --- | --- | --- | --- |
| **Cutoff ≥ 0.1 kUA/L** | 76.9% | 24.3% | 26.3% | 75.0% | 38% | 0.51 (0.37 - 0.64) |
| **Cutoff ≥ 0.35 kUA/L** | 61.5% | 43.2% | 27.6% | 76.2% | 48% | 0.52 (0.36 -0.68) |
| **Cutoff ≥ 0.7 kUA/L** | 53.8% | 51.4% | 28.0% | 76.0% | 52% | 0.53 (0.36 -0.69) |
| **Cutoff ≥ 3.5 kUA/L** | 15.4% | 86.5% | 28.6% | 74.4% | 68% | 0.51 (0.39 -0.63) |
| **Cutoff ≥ 4 kUA/L** | 15.4% | 94.6% | 50.0% | 76.1% | 74% | 0.55 (0.44-0.66) |
| **Cutoff ≥ 5 kUA/L** | 15.4% | 97.3% | 66.7% | 76.6% | 76% | 0.56 (0.46-0.67) |
| **Cutoff ≥ 6 kUA/L** | 15.4% | 97.3% | 66.7% | 76.6% | 76% | 0.56 (0.46-0.67) |
| **Cutoff ≥ 7 kUA/L** | 15.4% | 97.3% | 66.7% | 76.6% | 76% | 0.56 (0.46-0.67) |
| **Cutoff ≥ 8 kUA/L** | 15.4% | 97.3% | 66.7% | 76.6% | 76% | 0.56 (0.46-0.67) |
| **Cutoff ≥ 9 kUA/L** | 15.4% | 97.3% | 66.7% | 76.6% | 76% | 0.56 (0.46-0.67) |
| **Cutoff ≥ 10 kUA/L** | 7.7% | 97.3% | 50.0% | 75.0% | 74% | 0.52 (0.45-0.60) |

**Supplementary Table 2** Diagnostic accuracy of sIgE to tropomyosin at each cut-off point

|  | **Sensitivity (%)** | **Specificity (%)** | **PPV (%)** | **NPV (%)** | **Efficiency (%)** | **AUC** |
| --- | --- | --- | --- | --- | --- | --- |
| **Cutoff ≥ 0.1 kUA/L** | 23.1% | 83.8% | 33.3% | 75.6% | 68% | 0.53 (0.40-0.67) |
| **Cutoff ≥ 0.35 kUA/L** | 23.1% | 91.9% | 50.0% | 77.3% | 74% | 0.57 (0.45-0.70) |
| **Cutoff ≥ 0.7 kUA/L** | 15.4% | 94.6% | 50.0% | 76.1% | 74% | 0.55 (0.44 -0.66) |
| **Cutoff ≥ 3.5 kUA/L** | 7.7% | 100.0% | 100.0% | 75.5% | 76% | 0.54 (0.46-0.61) |
